# Supplementary material for: gjSOX9 Cloning, Expression, and Comparison with gjSOXs Family Members in Gekko japonicus
Source: Curr Issues Mol Biol. 2023 Nov 20;45(11):9328–41. doi: 10.3390/cimb45110584 (PMC10670703; doi:10.3390/cimb45110584)
Supplement: Supplementary file 1 [file cimb-45-00584-s001.zip › cimb-2703883-supplementary.pdf]

Table S1 gjSOX9 and its orthologs properties.

Table S2 gjSOX family members and their respective orthologs in this study.

Figure S1 Sequence alignment of gjSOX9

Figure S2 Hydrophilic analysis (left) and signal peptide prediction (right) of gjSOX9.

Figure S3 Phylogenetic analyses of gjSOX9 with its homologies.

Figure S4 Sequence conservation analyses of motif1-8 among 16 gjSOX members.

Figure S5 Multiple sequence alignment of HMGs for 16 gjSOX members.

Table S1 gjSOX9 and its orthologs properties. HI for hydrophilicity index; SL for subcellular localization. gjSOX9 sequence was cloned in this study while other SOX9 homologs sequences were retrieved from NCBI database.

Name	Accession	Species	Size	MW(kDa)	pI	HI*	SL*
gjSOX9	WGV33816.1	*Gekko japonicus*	494	55.35	6.31	-1.090	Nucleus
stSOX9	XP_048345639.1	*Sphacrodactylus townsendi*	495	55.04	6.20	-1.033	Nucleus
emSOX9	XP_054833382.1	*Eublepharis macularius*	494	55.23	6.31	-1.070	Nucleus
hcSOX9	XP_053156459.1	*Hemicordylus capensis*	498	55.75	6.31	-1.113	Nucleus
acSOX9	XP_003224717.1	*Anolis carolinensis*	526	57.36	6.26	-1.010	Nucleus
pbSOX9	XP_007429968.1	*Python bivittatus*	365	40.77	6.21	-1.140	Nucleus
zvSOX9	XP_034959970.1	*Zootoca vivipara*	499	55.55	6.31	-1.060	Nucleus
cmSOX9	XP_007056120.2	*Chelonia mydas*	480	53.63	6.23	-1.098	Nucleus
psSOX9	XP_025043574.1	*Pelodiscus sinensis*	494	55.09	6.31	-1.084	Nucleus
oaSOX9	XP_001506094.2	*Ornithorhynchus anatinus*	508	56.42	6.29	-1.107	Nucleus
clSOX9	NP_001002978.1	*Canis lupus familiaris*	513	56.56	6.60	-1.026	Nucleus
hsSOX9	NP_000337.1	*Homo sapiens*	509	56.14	6.31	-1.007	Nucleus
mmSOX9	NP_035578.3	*Mus musculus*	507	56.08	6.31	-1.021	Nucleus
xtSOX9	NP_001016853.1	*Xenopus tropicalis*	482	53.99	6.29	-1.110	Nucleus
drSOX9a	NP_571718.1	*Danio rerio*	462	51.33	5.98	-0.979	Nucleus
drSOX9b	NP_571719.1	*Danio rerio*	400	44.18	6.60	-0.918	Nucleus
olSOX9	NP_001098556.1	*Orizias latipes*	476	52.68	6.28	-0.963	Nucleus
tgSOX9	XP_030143168.3	*Taeniopygia guttata*	474	52.83	6.14	-1.078	Nucleus
ggSOX9	NP_989612.1	*Gallus gallus*	494	54.85	6.23	-1.078	Nucleus
mgSOX9	XP_010719808.1	*Meleagris gallopavo*	451	49.67	8.38	-1.019	Nucleus

Table S2 gjSOX family members and their respective orthologs in this study. The total of 150 SOX homologs from 7 species were utilized for phylogenetic analyses, including *G. japonicus* (*Gekko japonicus*) (16), *Z. vivipara* (*Zootoca vivipara*) (19), *X. laevis* (*Xenopus laevis*) (36), *T. scripta* (*Trachemys scripta elegans*) (18), *P. vitticeps* (*Pogona vitticeps*) (17), *M. musculus* (*Mus musculus*) (19) and *D.rerio* (*Danio rerio*) (25); Those SOX members were excluded among other 6 species, which had no orthologous relationships with any member of 16 gjSOX. HI for hydrophilicity index.

SOX	Family	Accession	Species	Size(aa)	MW(kDa)	HI*
gjSOX2	gjSOXB1	XP_015262440.1	*G. japonicus*	384	40.74	9.92
gjSOX3	gjSOXB1	XP_015267683.1	*G. japonicus*	318	34.01	9.62
gjSOX4	gjSOXC	XP_015271872.1	*G. japonicus*	420	44.34	6.74
gjSOX5	gjSOXD	XP_015268168.1	*G. japonicus*	764	84.30	6.12
gjSOX6	gjSOXD	XP_015277789.1	*G. japonicus*	787	87.40	6.54
gjSOX7	gjSOXF	XP_015284766.1	*G. japonicus*	375	41.46	6.16
gjSOX8	gjSOXE	XP_015279835.1	*G. japonicus*	325	35.64	6.28
gjSOX9	gjSOXE	WGV33816.1	*G. japonicus*	494	55.35	6.31
gjSOX10	gjSOXE	XP_015261463.1	*G. japonicus*	461	49.89	6.2
gjSOX11	gjSOXC	XP_015262936.1	*G. japonicus*	417	45.62	5.09
gjSOX12	gjSOXC	XP_015279104.1	*G. japonicus*	291	32.88	8.82
gjSOX13	gjSOXD	XP_015262805.1	*G. japonicus*	615	69.24	6.31
gjSOX14	gjSOXB2	XP_015276981.1	*G. japonicus*	240	26.64	9.68
gjSOX17	gjSOXF	XP_015282499.1	*G. japonicus*	177	18.63	5.94
gjSOX18L	gjSOXF	XP_015284209.1	*G. japonicus*	398	44.32	8.66
gjSOX30	gjSOXH	XP_015272912.1	*G. japonicus*	778	85.68	5.75
mmSOX1	mmSOXB1	NM_009233.3	*M. musculus*	391	39.05	9.7
mmSOX2	mmSOXB1	NM_011443.4	*M. musculus*	319	34.41	9.74
mmSOX3	mmSOXB1	NM_009237.2	*M. musculus*	450	45.44	9.78
mmSOX4	mmSOXC	NM_009238.3	*M. musculus*	440	45.04	7.15
mmSOX5	mmSOXD	XM_036165995.1	*M. musculus*	715	79.26	5.92
mmSOX6	mmSOXD	XM_030242296.2	*M. musculus*	787	87.32	6.95
mmSOX7	mmSOXF	NM_011446.1	*M. musculus*	380	41.49	6.01
mmSOX8	mmSOXE	XM_036160426.1	*M. musculus*	564	60.53	6.06
mmSOX9	mmSOXE	NM_011448.4	*M. musculus*	507	56.08	6.31
mmSOX10	mmSOXE	NM_011437.1	*M. musculus*	466	49.95	6.12
mmSOX11	mmSOXC	NM_009234.6	*M. musculus*	395	42.63	4.96
mmSOX12	mmSOXC	NM_011438.2	*M. musculus*	314	34.08	5.14
mmSOX13	mmSOXD	XM_030252397.2	*M. musculus*	613	68.17	6.03
mmSOX14	mmSOXD	NM_011440.1	*M. musculus*	240	26.52	9.68
mmSOX17	mmSOXF	NM_001289467.1	*M. musculus*	419	44.65	5.85
mmSOX18	mmSOXF	NM_009236.2	*M. musculus*	377	40.90	7.6
mmSOX21	mmSOXB2	NM_177753.3	*M. musculus*	276	28.61	9.74
mmSOX30	mmSOXH	XM_006532797.5	*M. musculus*	782	83.94	8.83
drSOX1a	drSOXB1	NP_001002483.1	*D. rerio*	336	36.26	9.7
drSOX1b	drSOXB1	NP_001032751.1	*D. rerio*	340	36.44	9.7
drSOX2	drSOXB1	NP_998283.1	*D. rerio*	315	34.70	9.74
drSOX3	drSOXB1	NP_001001811.2	*D. rerio*	300	33.42	9.63

drSOX4a	drSOXC	NP_998287.1	*D. rerio*	363	39.61	5.92
drSOX4b	drSOXC	NP_957195.1	*D. rerio*	342	36.96	7.77
drSOX5	drSOXC	NP_001028757.1	*D. rerio*	759	83.47	7.27
drSOX6	drSOXD	NP_001116481.1	*D. rerio*	768	85.79	6.56
drSOX7	drSOXF	NP_001074219.1	*D. rerio*	390	42.74	6.11
drSOX8a	drSOXE	NP_001271361.1	*D. rerio*	401	44.71	7.06
drSOX8b	drSOXE	NP_001020636.1	*D. rerio*	358	40.17	6.72
drSOX9a	drSOXE	NP_571718.1	*D. rerio*	462	51.33	5.98
drSOX9b	drSOXE	NP_571719.1	*D. rerio*	407	45.03	6.77
drSOX10	drSOXE	NP_571950.1	*D. rerio*	485	51.43	6.26
drSOX11a	drSOXC	NP_571411.1	*D. rerio*	354	39.92	5.34
drSOX11b	drSOXC	NP_571412.1	*D. rerio*	368	41.19	5.02
drSOX12	drSOXC	NP_001025449.1	*D. rerio*	355	39.93	6.09
drSOX13	drSOXD	NP_001352418.1	*D. rerio*	597	67.10	7.8
drSOX14	drSOXD	NP_001032769.1	*D. rerio*	238	26.55	9.68
drSOX17	drSOXF	NP_571362.2	*D. rerio*	413	45.90	6.92
drSOX18	drSOXF	XP_017209261.1	*D. rerio*	431	46.94	6.78
drSOX19a	drSOXB1	NP_570983.2	*D. rerio*	297	32.78	9.66
drSOX19b	drSOXB1	NP_571777.1	*D. rerio*	293	32.34	9.61
drSOX21b	drSOXB2	NP_001009888.1	*D. rerio*	245	26.80	9.74
drSOX32	drSOXK	NP_571926.1	*D. rerio*	307	35.32	8.41
tsSOX1	tsSOXB1	XM_034758144.1	*T. scripta*	366	37.81	9.7
tsSOX2	tsSOXB1	XR_004647093.1	*T. scripta*	315	34.50	9.74
tsSOX3	tsSOXB1	XM_034780494.1	*T. scripta*	314	33.50	9.66
tsSOX4	tsSOXC	XM_034762411.1	*T. scripta*	438	45.15	6.47
tsSOX5	tsSOXC	XM_034782427.1	*T. scripta*	791	86.96	6.23
tsSOX6	tsSOXD	XM_034770115.1	*T. scripta*	834	92.55	7.03
tsSOX7	tsSOXF	XM_034766720.1	*T. scripta*	381	42.04	6.35
tsSOX8	tsSOXE	XM_034783538.1	*T. scripta*	468	50.94	6.52
tsSOX9	tsSOXE	XM_034789829.1	*T. scripta*	492	54.92	6.29
tsSOX10	tsSOXE	XM_034776772.1	*T. scripta*	463	49.95	6.2
tsSOX11	tsSOXC	XM_034766450.1	*T. scripta*	399	44.40	4.94
tsSOX12	tsSOXC	XM_034787972.1	*T. scripta*	333	37.46	7.58
tsSOX13	tsSOXD	XM_034767283.1	*T. scripta*	616	69.44	6.46
tsSOX14	tsSOXB2	XM_034781927.1	*T. scripta*	240	26.65	9.68
tsSOX18	tsSOXF	XM_034787818.1	*T. scripta*	420	46.60	6.6
tsSOX21	tsSOXB2	XM_034758265.1	*T. scripta*	276	28.70	9.74
tsSOX30	tsSOXH	XM_034779823.1	*T. scripta*	569	61.84	6.6
pvSOX1	pvSOXB1	XP_020649967.1	*P. vitticeps*	160	17.88	10.34
pvSOX2	pvSOXB1	XP_020662416.1	*P. vitticeps*	320	34.60	9.74
pvSOX4	pvSOXC	XP_020637327.1	*P. vitticeps*	450	46.09	6.61
pvSOX5	pvSOXD	XP_020665637.1	*P. vitticeps*	770	85.11	6.12
pvSOX6	pvSOXD	XP_020658607.1	*P. vitticeps*	780	87.59	6.61
pvSOX7	pvSOXF	XP_020671506.1	*P. vitticeps*	370	40.93	6.57
pvSOX8	pvSOXE	XP_020661598.1	*P. vitticeps*	410	45.22	7.14
pvSOX9	pvSOXE	XP_020646934.1	*P. vitticeps*	500	56.08	6.32
pvSOX10	pvSOXE	XP_020643402.1	*P. vitticeps*	460	49.92	6.20
pvSOX11	pvSOXC	XP_020638248.1	*P. vitticeps*	440	47.92	4.87

pvSOX12	pvSOXC	XP_020670327.1	*P. vitticeps*	320	36.31	8.25
pvSOX13	pvSOXD	XP_020652686.1	*P. vitticeps*	610	69.53	6.34
pvSOX14	pvSOXB2	XP_020643117.1	*P. vitticeps*	240	26.68	9.68
pvSOX17	pvSOXF	XP_020651605.1	*P. vitticeps*	250	27.87	8.34
pvSOX18	pvSOXF	XP_020670054.1	*P. vitticeps*	370	41.85	8.18
pvSOX21	pvSOXB2	XP_020639150.1	*P. vitticeps*	189	21.51	9.83
pvSOX30	pvSOXH	XP_020647023.1	*P. vitticeps*	777	85.17	6.66
zvSOX1	zvSOXB1	XP_034971802.1	*Z. vivipara*	395	39.76	9.7
zvSOX2	zvSOXB1	XP_034988520.1	*Z. vivipara*	329	35.71	9.74
zvSOX3	zvSOXB1	XP_034981651.1	*Z. vivipara*	337	35.66	9.61
zvSOX4	zvSOXC	XP_034981245.1	*Z. vivipara*	432	44.45	6.68
zvSOX5	zvSOXD	XP_034983770.1	*Z. vivipara*	809	89.17	6.88
zvSOX6	zvSOXD	XP_034975806.1	*Z. vivipara*	791	87.87	6.69
zvSOX7	zvSOXF	XP_034966760.1	*Z. vivipara*	379	41.63	6.32
zvSOX8	zvSOXE	XP_034987881.1	*Z. vivipara*	476	51.64	6.56
zvSOX9	zvSOXE	XP_034959970.1	*Z. vivipara*	504	56.21	6.32
zvSOX10	zvSOXE	XP_034984118.1	*Z. vivipara*	466	50.53	6.21
zvSOX11	zvSOXC	XP_034965784.1	*Z. vivipara*	431	46.75	5.03
zvSOX12	zvSOXC	XP_034979381.1	*Z. vivipara*	330	37.03	7.81
zvSOX13	zvSOXD	XP_034978663.1	*Z. vivipara*	620	69.65	5.96
zvSOX14	zvSOXB2	XP_034989497.1	*Z. vivipara*	245	27.36	9.68
zvSOX17	zvSOXF	XP_034981552.1	*Z. vivipara*	397	42.84	6.19
zvSOX18	zvSOXF	XP_034977958.1	*Z. vivipara*	464	50.46	6.9
zvSOX21	zvSOXB2	XP_034972104.1	*Z. vivipara*	290	30.29	9.74
zvSOX30	zvSOXH	XP_034961649.1	*Z. vivipara*	787	86.54	6.60
xlSOX1.L	xlSOXB1	XP_018102680.1	*X. laevis*	400	41.42	9.7
xlSOX1.S	xlSOXB1	NP_001089143.1	*X. laevis*	390	41.06	9.65
xlSOX2.L	xlSOXB1	NP_001081691.1	*X. laevis*	310	34.11	9.74
xlSOX3.L	xlSOXB1	NP_001166875.1	*X. laevis*	300	33.83	9.66
xlSOX3.S	xlSOXB1	NP_001084148.1	*X. laevis*	300	33.83	9.66
xlSOX4.L	xlSOXC	NP_001079998.1	*X. laevis*	380	41.34	6.38
xlSOX4.S	xlSOXC	NP_001090179.1	*X. laevis*	370	40.33	6.39
xlSOX4	xlSOXC	NP_001165672.1	*X. laevis*	370	40.46	6.57
xlSOX4-1	xlSOXC	NP_001091911.1	*X. laevis*	370	40.35	6.39
xlSOX5.L	xlSOXD	XP_018107319.1	*X. laevis*	750	83.97	6.85
xlSOX5.S	xlSOXD	NP_001167508.1	*X. laevis*	650	72.46	8.84
xlSOX6.S	xlSOXD	NP_001267587.1	*X. laevis*	760	84.86	7.26
xlSOX7.L	xlSOXF	NP_001079337.1	*X. laevis*	360	40.87	6.01
xlSOX7.S	xlSOXF	XP_018121508.1	*X. laevis*	360	41.12	6.1
xlSOX8.L	xlSOXE	NP_001083964.1	*X. laevis*	450	50.47	6.59
xlSOX8.S	xlSOXE	XP_018094788.1	*X. laevis*	460	50.52	6.59
xlSOX9.L	xlSOXE	NP_001087942.1	*X. laevis*	470	53.32	6.29
xlSOX9.S	xlSOXE	NP_001084276.1	*X. laevis*	470	53.38	6.35
xlSOX10.L	xlSOXE	NP_001082358.1	*X. laevis*	440	49.10	5.63
xlSOX10.S	xlSOXE	XP_018116740.1	*X. laevis*	430	48.21	6.12
xlSOX11.L	xlSOXC	NP_001135834.1	*X. laevis*	380	42.73	4.87
xlSOX11.S	xlSOXC	NP_001084325.1	*X. laevis*	370	42.09	4.93
xlSOX12.L	xlSOXC	XP_018090156.1	*X. laevis*	250	28.07	8.62

xlSOX12.S	xlSOXC	NP_001079045.1	*X. laevis*	240	27.28	8.46
xlSOX13.S	xlSOXD	NP_001081238.1	*X. laevis*	560	63.41	6.21
xlSOX14.L	xlSOXB2	XP_018119464.1	*X. laevis*	230	26.84	9.68
xlSOX14.S	xlSOXB2	NP_001165685.1	*X. laevis*	230	26.86	9.68
xlSOX17a.L	xlSOXF	NP_001081631.1	*X. laevis*	380	42.65	6.24
xlSOX17a.S	xlSOXF	NP_001083136.1	*X. laevis*	370	42.52	6.11
xlSOX17b.1.L	xlSOXF	NP_001092185.1	*X. laevis*	370	43.12	5.82
xlSOX17b.1.S	xlSOXF	NP_001081633.1	*X. laevis*	370	43.26	5.76
xlSOX18.L	xlSOXF	NP_001082102.1	*X. laevis*	360	40.95	6.87
xlSOX18.S	xlSOXF	NP_001082104.1	*X. laevis*	360	40.58	6.43
xlSOX21.L	xlSOXB2	XP_018102738.1	*X. laevis*	270	28.48	9.74
xlSOX21.S	xlSOXB2	NP_001165684.1	*X. laevis*	260	28.12	9.68

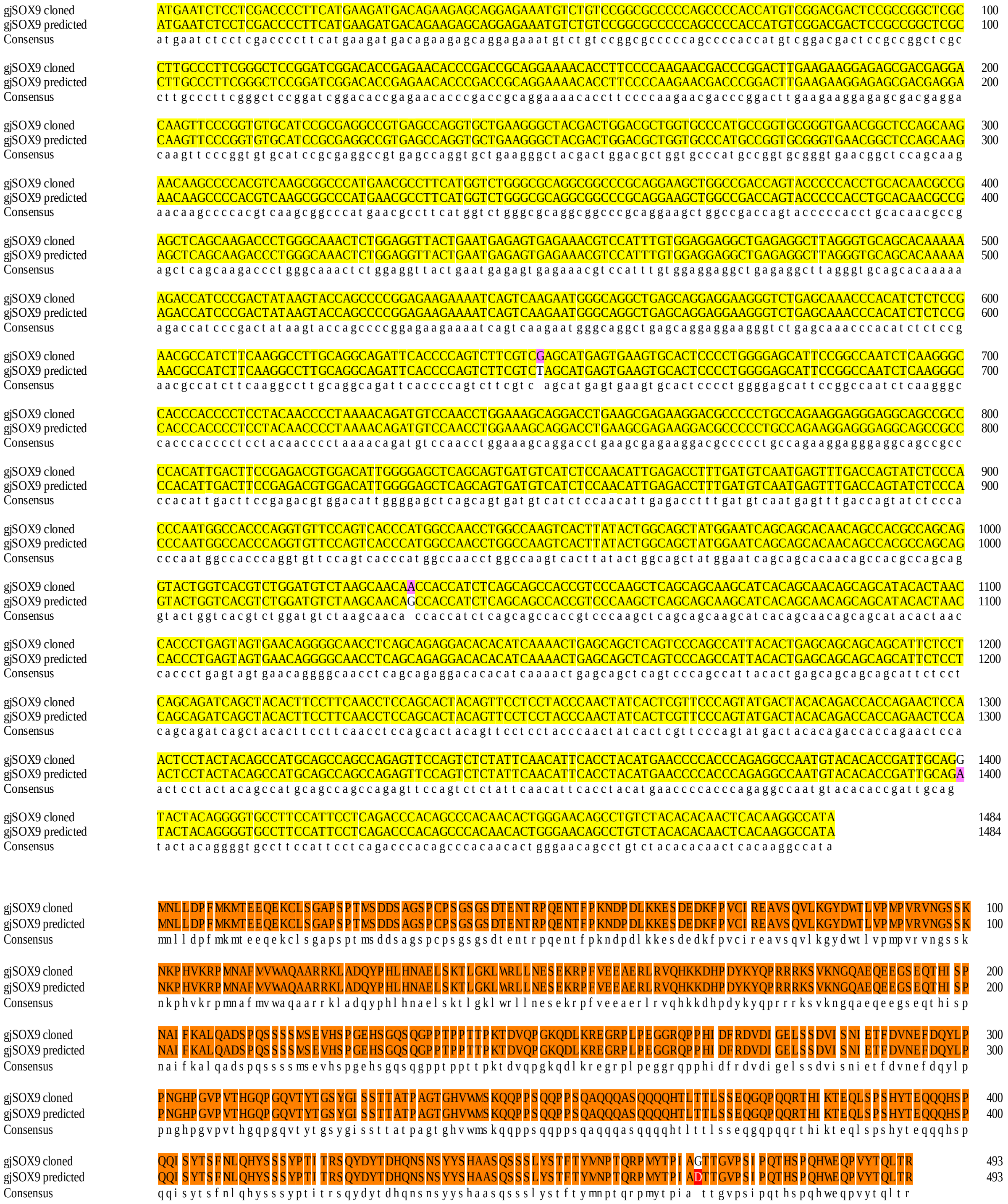

Figure S1 Sequence alignment of gjSOX9. The upper for nucleotides sequence alignment of gjSOX encoding region between cloned cDNA (OQ935362) in this study and annotation sequence from *G. japonicus* genome (XM_015422341), and the lower for amino acids sequence alignment between deduced amino acids sequence from cloned cDNA in this study (WGV33816.1) and from *G. japonicus* genome (XP_015277827.1). Sequence alignment was performed by DNAMAN9.0. The red "letter" means different nucleotide or amino acid residue.

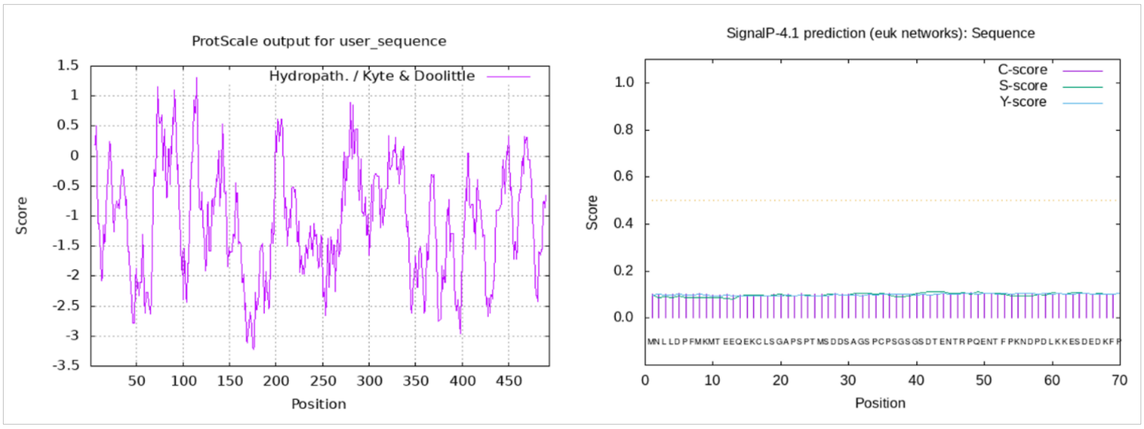

Figure S2 Hydrophilic analysis (left) and signal peptide prediction (right) of gjSOX9. Hydrophilic analysis was performed by online software protScale and signal peptide was predicted using online software SingalP4.1.

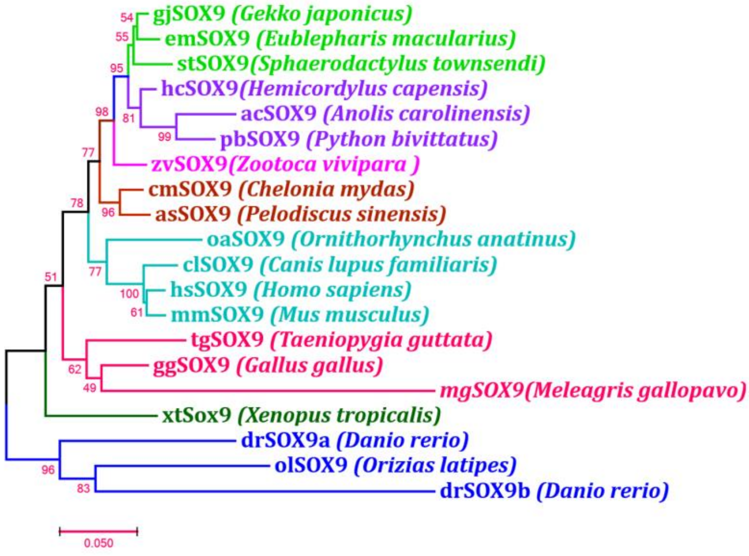

Figure S3 Phylogenetic analyses of gjSOX9 with its homologies. The phylogenetic tree was reconstructed using MEGA11.0 software by neighbor-joining method with1000 bootstrap replicates based on amino acid sequence alignment. Sequence information of gjSOX9 and its orthologs were the same as those in Table S1

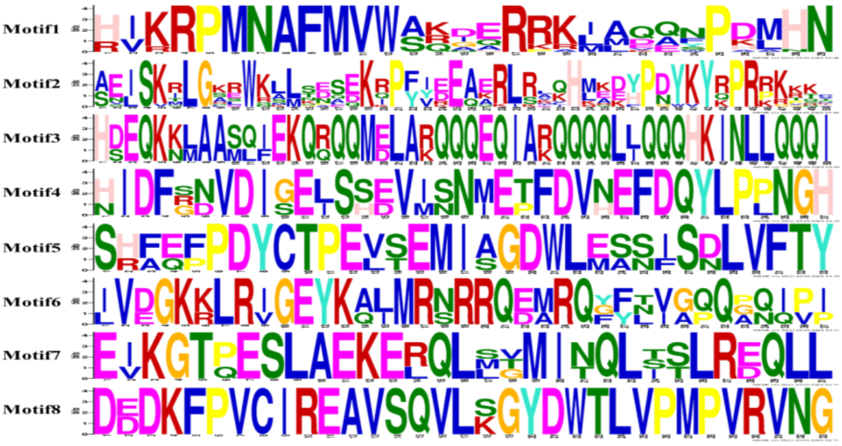

Figure S4 Sequence conservation analyses of Motif 1-8 among 16 gjSOX members. Motifs sequence conservation were analyzed by online MEME software. A sequence logo consists of a stack of letters at each position. The relative sizes of the letters indicate their frequency in the sequences.

gjSOX2	RVKRPMNAFMVWSRGQRRKMAQENPKMHNSEISKRLGAEWKLLSEAEKRPFIDEAKRLRALHMKEHPDYKYRPRRKTKT	79
gjSOX3	RVKRPMNAFMVWSRGQRRKMAQENPKMHNSEISKRLGADWKLLSDSEKRPFIDEAKRLRAVHMKEYPDYKYRPRRKTKT	79
gjSOX4	HIKRPMNAFMVWSQIERRKIMEQSPDMHNAEISKRLGKRWKLLKDSDKIPFIREAERLRLKHMADYPDYKYRPRKKVKS	79
gjSOX5	LLAMGSG.NFGEIKGTPESLAEKERQLM.GMNQLTSLREQLLAAHDEQKKLAASQIEKQRQQMELAKQQQEQIARQQQ	77
gjSOX6	MERLNSSELLGEIKGTPESLAEKERQLS.TMTQLISLREQLLAAHDEQKKLAASQIEKQRQQMDLARQQQEQIARQQQ	78
gjSOX7	RIRRPMNAFMVWAKDERKRLAVCNPDLHNAELSKMLGKSWKALTPSQKRPYVEEAERLRVQHMRDYPNYKYRPRRKKQV	79
gjSOX8	....LLSESEKRPFVEEAERLRVQHKKDHPDYKYQPRRRKSV	38
gjSOX9	HVKRPMNAFMVWAQAARRKLADQYPHLHNAELSKTLGKLWRLLNESEKRPFVEEAERLRVQHKKDHPDYKYQPRRRKSV	79
gjSOX10	HVKRPMNAFMVWAQAARRKLADQYPHLHNAELSKTLGKLWRLLNESDKRPFIEEAERLRMQHKKDHPDYKYQPRRRKNG	79
gjSOX11	HIKRPMNAFMVWSKIERRKIMEQSPDMHNAEISKRLGKRWKMLKDSEKIPFIREAERLRLKHMADYPDYKYRPRKKPKI	79
gjSOX12	HIKRPMNAFMVWSQNERRKIMDQWPDMHNAEISKRLGRRWQLLQDSEKIPFVKEAERLRLKHMADYPDYKYRPRKKGKM	79
gjSOX13	LLGKSPT.ESKEVKGTQESLAEKELQLL.VMNQLSTLRDQLLTAHSEQKNMAAMLFEKQQQQMELARQQQEQIAKQQQ	77
gjSOX14	HIKRPMNAFMVWSRGQRRKMAQENPKMHNSEISKRLGAEWKLLSEAEKRPYIDEAKRLRAQHMKEHPDYKYRPRRKPKN	79
gjSOX17	RIRRPMNAFMVWAKDERKRLAQCNPDLHNAELSKMLGKSGREHQSGTTAARLGKPLFALLADVDRAEFEQYLPFGCRPA	79
gjSOX18L	PFTR..ATRVPCPERPRKRLAQCNPDLHNAVLSKMLGQSWKALGASDKRPFVEEAERLRIQHLQDHPNYKYRPRRKKQA	77
gjSOX30	HVKRPMNAFMVWARIHRPALAKANPAANNAEISVQLGLEVNKLTEEQKKPYYDEAQKIKEKHREEFPGWVYQPRPGKRK	79

Figure S5 Multiple sequence alignment of HMGs for 16 gjSOX members. Alignment analysis was performed by DNAMAN9.0. The red "letter" means the sequence identity≥75% while yellow "letter "means sequence identity≥50%.
